# Acute Prolonged Hamstrings Vibration Reduces Limb Stiffness Following Anterior Cruciate Ligament Reconstruction During a Single‐Limb Drop‐Jump Task

**DOI:** 10.1002/jor.26105

**Published:** 2025-05-25

**Authors:** Timothy Lowe, Hao‐Yuan Hsiao, Xuanliang Neil Dong, Lisa Griffin

**Affiliations:** ^1^ Department of Kinesiology and Health Education The University of Texas at Austin Austin Texas USA; ^2^ Department of Health and Kinesiology The University of Texas at Tyler Tyler Texas USA

**Keywords:** kinematics, kinetics, knee, limb loading, osteoarthritis

## Abstract

Impaired quadriceps function influences lower limb biomechanics following anterior cruciate ligament reconstruction (ACLR). This often results in stiff limb loading which leads to the development of knee osteoarthritis. Greater hamstrings/quadriceps co‐activation is common after ACLR and is, in part, responsible for impaired quadriceps function. Prolonged vibration of the hamstrings can alleviate reciprocal inhibition of the quadriceps and enhance quadriceps activation. We hypothesize that this will also reduce limb stiffness. Fourteen participants with unilateral ACLR, and 14 non‐injured individuals performed a single‐leg drop‐landing task, before and after 20 min of hamstrings vibration. Limb stiffness, peak vertical ground reaction force, peak instantaneous loading rate, knee excursion, and peak knee extension moment were calculated during the loading phase of the drop‐landing task. The ACLR group had significantly greater limb stiffness (*p* = 0.002), peak vertical ground reaction force (*p* = 0.004), loading rate (*p* = 0.001), significantly less knee excursion (*p* = 0.009) and knee extension moment (*p* = 0.013) before vibration than non‐injured controls. Vibration significantly reduced limb stiffness (*p* = 0.001), peak vertical ground reaction force (*p* = 0.001), loading rate (*p* < 0.001), significantly increased knee excursion (*p* = 0.01) and knee extension moment (*p* < 0.001) in the ACLR group. No significant differences were found following vibration in the non‐injured control group.

**Clinical Significance:**

These results demonstrate that prolonged vibration of the hamstrings has the potential to mitigate the stiff limb loading strategy linked to knee osteoarthritis development, and may represent an effective adjunct therapy for ACLR rehabilitation.

## 1 Introduction

Individuals who have undergone anterior cruciate ligament reconstruction (ACLR) demonstrate persistent movement differences compared to individuals without a history of ACLR. Kinetic and kinematic differences have been identified during walking [1], running [2], double‐leg landing tasks [3] and single‐leg landing tasks [4] when compared to both the uninvolved limb and to non‐injured individuals. These differences persist for years and present risk factors for a second ACL injury [5] and the development of osteoarthritis (OA) [6]. Task differences post‐ACLR include reduced knee flexion excursion during jump landing and single‐leg landing [7, 8], decreased knee extension moments (KEM) during landing tasks [3, 8], increased peak vertical ground reaction force (vGRF) during jump and single‐leg landings [7, 8] and loading rates during walking, single‐leg step down and landing tasks [9, 10]. Limb stiffness is characterized by reduced knee excursion and increased peak forces and loading rates [11]. These observations suggest that a stiffer limb‐loading strategy exposes the knee to higher joint forces, increasing risk of reinjury [5], and development of knee OA [6]. Stiff limb loading is linked to reinjury, altered cartilage morphology [12] and radiographic evidence of early degeneration of knee joint cartilage in individuals with ACLR [13, 14].

Quadriceps weakness and decreased ability to voluntarily activate the quadriceps are common after ACLR [15] and patients with diminished quadriceps function adopt a stiffer limb‐loading strategy because of an inability of the quadriceps to sufficiently control knee joint motion and attenuate force [6, 16]. Suggesting that poor quadriceps function may result in altered sagittal‐plane knee biomechanics during walking and landing [17, 18, 19, 20]. Moreover, the quadriceps are imperative for both generating and attenuating force about the knee during dynamic activities. Landing from a jump is a highly dynamic physical task that requires the development of a sufficient internal KEM, generated by eccentric action of the quadriceps to control knee joint motion and attenuate impact forces [21]. Secondary ACL injury risk appears to be strongly related sagittal‐plane knee moments during jump landing [22, 23].

Much of the focus regarding the interplay between biomechanics and articular cartilage composition has focused on the influence of low‐impact, cyclical loading such as walking gait [24, 25]. However, individuals who sustain ACL injuries are often young and physically active and engage in dynamic movements such as jump landing more commonly in their daily activities. Moreover, lower peak KEM and greater peak vGRF during jump landing have been shown to be related to longer T1r relaxation times, suggesting worse articular cartilage composition, and greater risk of OA [12]. Therefore, examining jump landings may provide insight not only into reinjury risk but OA risk as well.

Limb stiffness is modifiable and may be increased or decreased with footwear [26], contact surface [27], verbal cues [28, 29, 30, 31], and training [32, 33]. Quadriceps weakness following ACLR is due, in part, to an ongoing neural inhibition of the quadriceps [15] due to diminished spinal reflexive inputs [34, 35] and cortical motor drive [36, 37] and increased hamstrings activity [38, 39]. Increased hamstrings activity creates greater co‐activation of the hamstrings and quadriceps [40], and increases reciprocal inhibition to the quadriceps [41, 42]. Using prolonged vibration of the hamstrings to fatigue the muscle spindles, we previously demonstrated that vibration reduces hamstrings/quadriceps co‐activation and enhances voluntary activation of the quadriceps in patients post‐ACLR [39, 43], suggesting that prolonged hamstrings vibration alleviates reciprocal inhibition of the quadriceps. Increasing voluntary activation capacity increases quadriceps maximal force output [16] and reduces limb stiffness (lower vGRF loading rate, greater knee excursion and KEM) [44]. Therefore, prolonged hamstrings vibration may be used to modify limb stiffness.

In the present study, we aimed to determine the acute effects of a single bout of prolonged hamstrings vibration on limb stiffness in patients post‐ACLR with matched non‐injured controls. We hypothesized that prolonged hamstrings vibration would alter limb biomechanics consistent with lower reinjury and OA risks [6] (i.e., increases in knee excursion and KEM, and decreases in peak vGRF and loading rates) in individuals with ACLR.

## 2 Methods

### 2.1 Participants

A minimum sample size (*n* = 20) was determined from power analysis using *α* = 0.05, *β* = 0.20 (power = 0.8), and effect size 0.53, based on our previous work [39, 45] for this prospective study (level II). Fourteen individuals who had undergone primary unilateral ACLR, and 14 non‐injured (NI) individuals with no history of injury or surgery to either leg, participated in this study (Table 1). Individuals in the ACLR group were a minimum of 6 months post‐surgery and were cleared by their physician to return to physical activity. We excluded individuals with bilateral ACLR, ACLR revision surgery, pregnancy, or any medical condition that would prevent them from performing the task described below. Age, sex, graft type, concomitant injury/procedure and date of ACLR were self‐reported. Level of physical activity was assessed using the Tegner Activity Scale [46], and self‐reported disability was assessed via the subjective section of the International Knee Documentation Committee Scale (IKDC) [47]. Participants in the NI group were matched to the ACLR group for age, sex, and physical activity level based on group averages. The study was approved by the Institutional Review Board at the University of Texas at Austin. All participants provided written informed consent before participation.

**Table 1:** Participant characteristics (mean ± SE) of ACLR and NI groups.

	ACLR	NI
*n*	14	14
Age (yrs)	26.6 ± 1.9 (18–39)	26.9 ± 1.3 (20–39)
Sex	7 M, 7 F	8 M, 6 F
Body mass (Kg)	74.2 ± 3.7 (56.6–106.5)	72.2 ± 3.4 (50.8–97.5)
Body height (m)	1.73 ± 0.02 (1.56–1.88)	1.71 ± 0.02 (1.52–1.85)
Graft type	Patella, 11	—
	Allograft, 3	—
Time post ACLR (months)	16.5 ± 1.5 (6–24)	—
IQR	9.75	—
Concomitant injury/procedure		
None	6	—
Lateral meniscus	5	—
Medial meniscus	1	—
MCL	2	—
Limb	6 L, 8 R	4 L, 10 R
Central activation ratio	88.3 ± 3.1^a^ (82–93)	98.0 ± 0.9 (97–99)
Tegner	7.3 ± 0.5 (6.1–8.5)	7.2 ± 0.3 (6.3–8.7)
IKDC	86.5 ± 2.5^a^ (73.3–88.9)	98.6 ± 0.3 (93.1–99.4)

a Indicates significant group difference, *p* < 0.05.

Abbrevations: IKDC, International Knee Documentation Committee; IQR, interquartile range; Kg, kilograms; MCL, medial collateral ligament; m, meters; yrs, years.

### 2.2 Experimental Setup

The data presented in this study are a secondary analysis of data that has been published previously [43]. Two experimental sessions were attended at least 48 h apart. During the first session, participants were familiarized with the testing protocol, provided consent, and were screened for inclusion/exclusion criteria. Voluntary activation of the quadriceps was assessed with the central activation ratio (CAR) using the superimposed burst technique [48]. During the second session, lower limb biomechanics were evaluated during a single‐leg drop‐landing task. The testing sequence was as follows: (1) warm‐up, (2) practice trials, (3) three successful trials, (4) vibration intervention, followed immediately by (5) three successful trials. The ACLR limb was assessed for ACL participants. Limb preference was noted as the limb preferred to initiate movement, and if the preferred limb was injured, a matched NI participant was assessed on the preferred limb. If the non‐preferred limb was injured, the non‐preferred limb of the NI participant was assessed.

### 2.3 Single‐Leg Drop‐Jump Task

Lower extremity kinematic and kinetic data were calculated using 3‐dimensional motion analysis during a single‐leg drop vertical jump task onto a force plate (Bertec Corp., Columbus, OH). Participants were outfitted with 39 retroreflective markers attached to anatomic landmarks according to the Vicon Full Body Plug‐in Gait marker set. To complete the single‐leg drop‐land task, participants stood at the edge of a 30‐cm box on the limb being tested and were instructed to drop off of the box and land on a force plate on the same limb then immediately jump upward as high as possible. Each participant performed a 5‐min warm‐up exercise on a cycle ergometer (Monark 828E). Participants were then familiarized with the protocol and allowed a maximum of five practice trials (range: 3–5). Then each performed three successful trials. A trial was considered successful if the participant landed with the whole foot on the force plate, jumped straight up, and landed again with the whole foot on the force plate, and maintained balance without taking an extra step or placing the contralateral limb down for 3 s. If a trial was unsuccessful, a subsequent trial was collected for analysis. No specific instructions were provided regarding arm position or landing technique. Following vibration another three successful trials were recorded for analysis. The total attempted trials ranged from 5 to 7 (successful + unsuccessful trials).

### 2.4 Prolonged Muscle Vibration

Each participant underwent a single session of prolonged hamstrings vibration. In a seated position, a 30 Hz vibration was applied directly to the hamstrings at rest for 20 min using a Thumper Versa Pro Massager (Thumper Massager Inc., Markham, ON) with the hips and knees flexed [39, 45].

### 2.5 Data Analysis

Kinematic data were recorded using a 12‐camera motion capture system (Vicon Motion Systems, Centennial, CO), sampled at 100 Hz [49]. Kinetic data were recorded at 2,000 Hz from a force plate embedded in the floor. Kinematic and kinetic data were digitally low‐pass filtered at 5 and 12 Hz, respectively, using a fourth order zero‐lag Butterworth filter [49, 50, 51].

**Figure 1:**
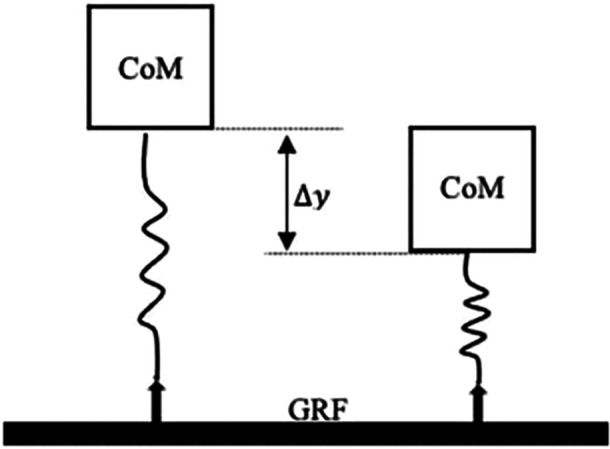
An example of the simple spring‐mass model used to approximate lower limb stiffness [53]. Spring‐mass model used for calculating stiffness when the leg is oriented vertically (i.e. hopping or jumping). CoM = center of mass, GRF = ground reaction force, ∆y = center of mass displacement, L_0_ = leg length.

Lower extremity biomechanics were evaluated over the loading phase of the initial landing, which was defined as the interval from initial contact (the instant vGRF > 20 N) to peak knee flexion [52]. Limb stiffness was calculated as:

$$k_{\mathrm{vert}}=F_{\max}/∆y$$

where F_max_ = maximum vGRF; ∆y = maximum vertical displacement of the center of mass (Figure 1). Knee flexion excursion was calculated for each trial by subtracting the joint angle at initial contact from the peak knee flexion angle during the loading phase. Peak vGRF was normalized to body weight (xBW). The peak instantaneous loading rate was calculated as the 1st‐time derivative of the vGRF (xBW*s^−1^). Knee joint moments were calculated using an inverse‐dynamics approach [54]. Internal KEM was normalized by dividing the product of body weight and height (xBW * Ht). All ﻿data were processed using a custom MATLAB program (MathWorks, Natick, MA).

### 2.6 Statistical Analysis

Statistical analyses were performed with SPSS software (Version 27, Chicago, IL). All descriptive statistics presented in the text, tables and figures are mean values ± SE. Data normality was verified using the Shapiro‐Wilk normality test and visual inspection of qq plots. A mixed‐model ANOVA was used to compare Group (2 levels) × Time (2 levels) differences. The dependent variables were: stiffness, peak vGRF, peak loading rate, knee excursion, and KEM. Independent variables included: Group (ACLR *vs.* NI) as a between‐subjects factor, and Time (pre‐ *vs.* post‐vibration) as a within‐subjects factor. When the ANOVA identified significant effects, Bonferroni corrections for post hoc comparisons was used. Regression was used to evaluate the association between baseline CAR and time since ACLR with the magnitude of change in peak stiffness, vGRF, loading rate, knee excursion, and KEM from pre‐ to post‐vibration in ACLR participants. Magnitude of change was calculated by subtracting the pre‐ from the post‐vibration value. A positive value indicated and increase after vibration and a negative value indicated a decrease following vibration. Statistical significance was set at *p* < 0.05.

## 3 Results

### 3.1 Limb Stiffness

**Figure 2:**
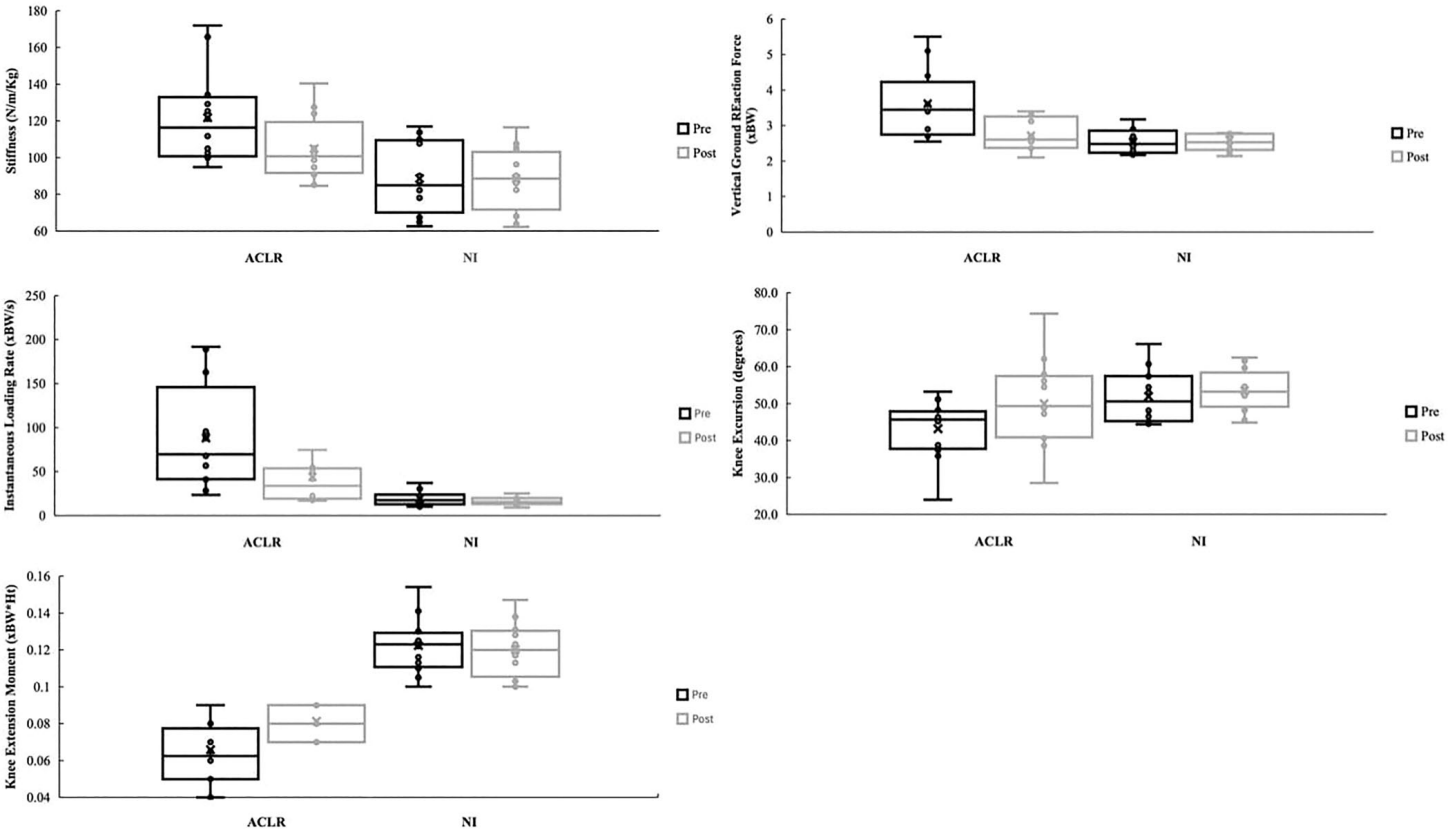
Descriptive statistics of (a) stiffness, (b) vGRF, (c) instantaneous loading rate, (d) knee flexion excursion and (e) knee extension moment during the deceleration phase of the drop‐land task pre‐ and post‐vibration. *Indicates significant difference, *p* < 0.05.

There was a significant interaction effect for Time and Group [*F* (1, 22) = 6.73, *p* = 0.02]. There was a significant main effect of Group [*F* (1, 22) = 10.8, *p* = 0.003], a significant main effect of Time [*F* (1, 22) = 6.893, *p* = 0.02]. Post‐hoc analysis revealed that the ACLR group had significantly greater stiffness pre‐ (*p* = 0.002) and post‐vibration (*p* = 0.03) than the NI group. Vibration significantly reduced stiffness in the ACLR group (*p* = 0.001), but had no effect on the NI group (*p *= 1.0) Additionally, there was no post‐vibration differences between groups (Figure 2a).

### 3.2 Peak Vertical Ground Reaction Force

There was a significant interaction effect for Time and Group [*F* (1, 22) = 20.4, *p* < 0.001], a significant main effect of Group [*F* (1, 22) = 8.0, *p* = 0.01], and a significant main effect of Time [*F* (1, 22) = 15. 7, *p* = 0.001]. Post‐hoc analysis revealed that the ACLR group landed with greater peak vGRF than the NI group pre‐vibration (*p* = 0.001). Vibration significantly lowered the peak vGRF in the ACLR group (*p* < 0.001), but had no effect on the NI group (*p *= 0.7); moreover there was no post‐vibration group difference (Figure 2b).

### 3.3 Instantaneous Loading Rate

There was a significant interaction effect of Group and Time [*F* (1, 11) = 10.9, *p* = 0.003], a significant main effect of Group [*F* (1, 22) = 13.8, *p* = 0.001], and a significant main effect of Time [*F* (1, 22) = 14.1, *p* = 0.001]. Post‐hoc analysis revealed that the ACLR group landed with a significantly higher loading rate pre‐ (*p* = 0.001) and post‐ (*p* = 0.01) vibration than the NI group. Vibration significantly reduced the loading rate in the ACLR group (*p* < 0.001), but had no effect on the NI group (*p *= 0.8, Figure 2c).

### 3.4 Knee Excursion

There was a significant interaction effect of Time and Group [*F* (1, 22) = 3.8, *p* = 0.05]. There was no main effect of Group [*F* (1, 22) = 3.5, *p* = 0.07]. However, there was a significant main effect of Time [*F* (1, 22) = 9.7, *p* = 0.005]. Post‐hoc analysis revealed that the ACLR group had less knee excursion pre‐vibration than the NI group (*p* = 0.01). Vibration increased knee excursion in the ACLR group (*p* = 0.002), but had no effect on the NI group (*p *= 0.4). Post‐vibration there were no group differences(Figure 2d).

### 3.5 Knee Extension Moment

**Figure 3:**
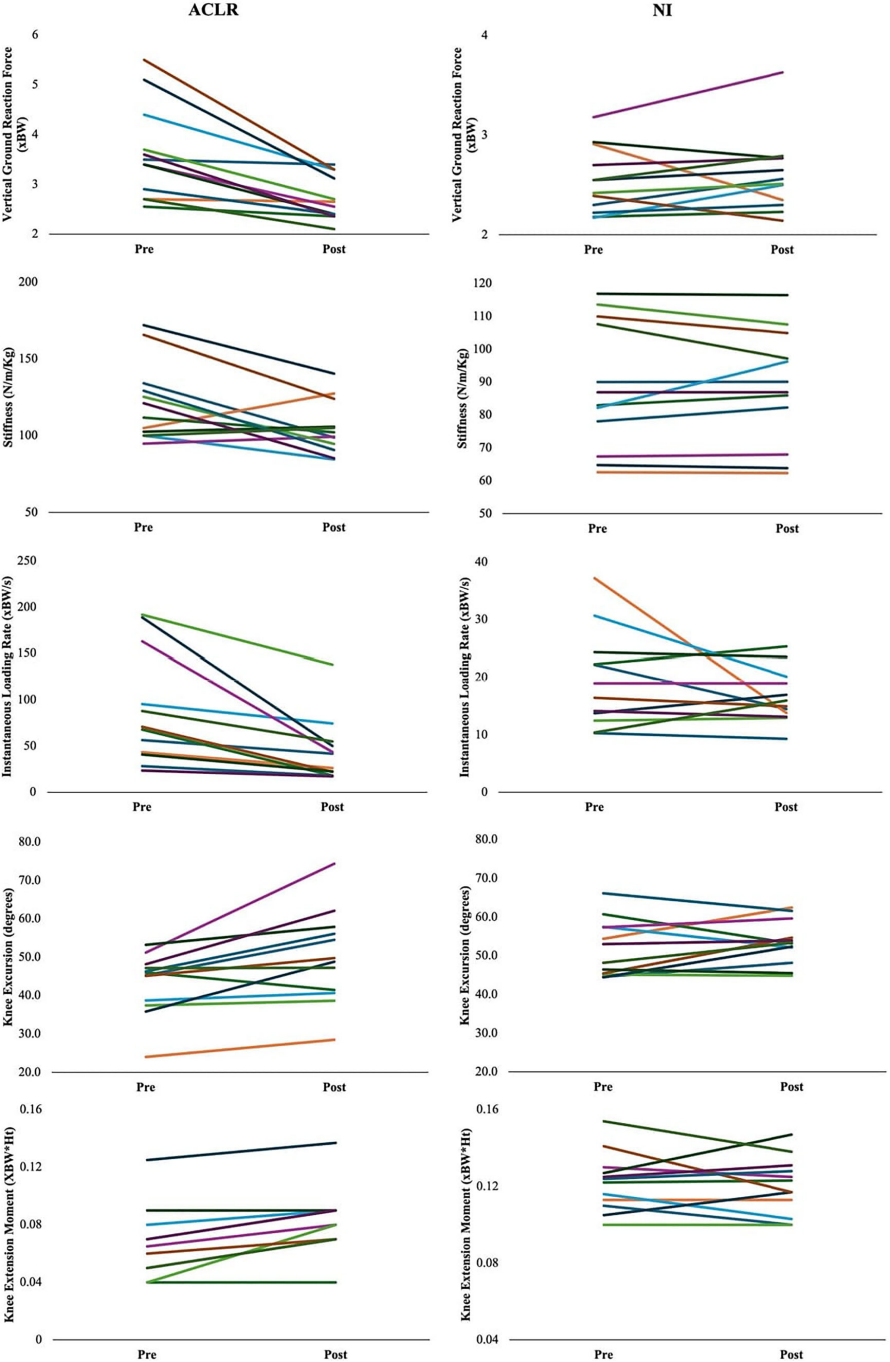
Descriptive statistics of the magnitude of change for (a) vGRF, (b) stiffness, (c) instantaneous loading rate, (d) knee flexion excursion and (e) knee extension moment during the deceleration phase of the drop‐land task pre‐ and post‐vibration in ACLR and NI groups.

There was a significant interaction effect of Group and Time [*F* (1, 22) = 13.2, *p* = 0.001], a significant main effect of Group [*F* (1, 22) = 72.0, *p* < 0.001], and a significant main effect of Time [*F* (1, 22) = 7.8, *p* = 0.01], and Post‐hoc analysis revealed that the ACLR group had smaller KEM pre‐ (*p* = 0.01) and post‐ (*p* = 0.01) vibration than the NI group. Vibration significantly increased KEM in the ACLR group (*p* < 0.001), but had no effect on the NI group *p *= 0.6, Figure 2e). (Figure 3).

### 3.6 Effect of Time Since Surgery and Baseline Central Activation Ratio on the Magnitude of Change

**Figure 4:**
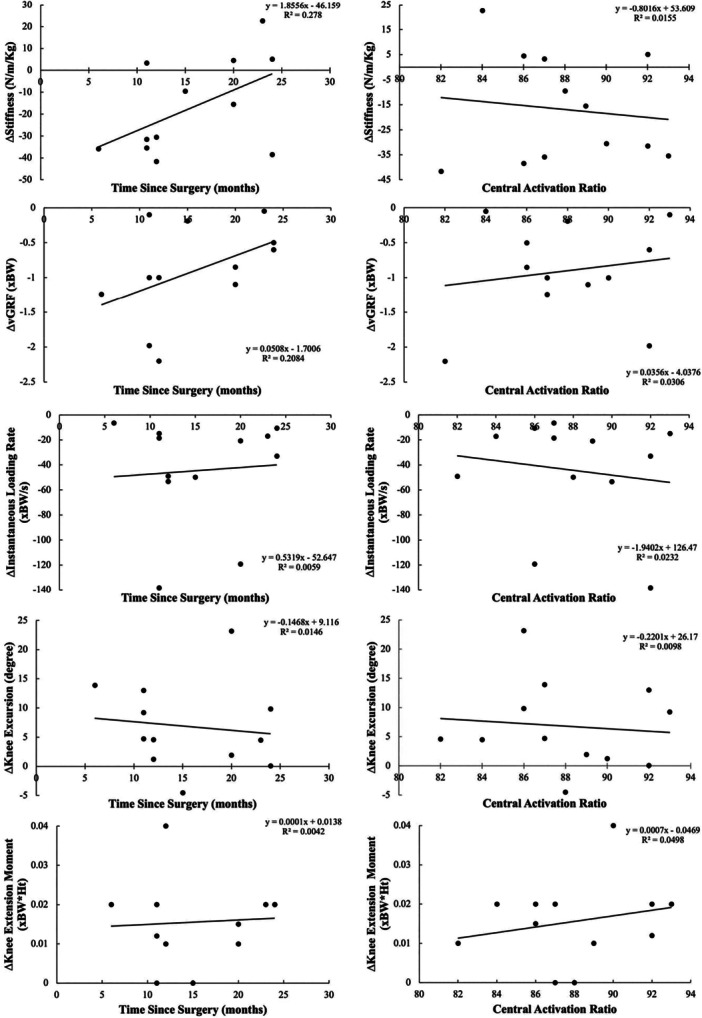
Regression analysis of time since ACLR surgery (months) and baseline CAR with the change in stiffness, peak vGRF, peak loading rate, knee excursion, and KEM from pre‐ to post‐vibration in ACLR participants.

There was a significant positive association between time since surgery and the magnitude of change in stiffness (R^2^ = 0.278, *p* = 0.02, *β *= 1.86, Figure 4). For every month post‐ACLR the effect of vibration on stiffness decreased by 1.86 N/m/Kg, suggesting that the effect of vibration decreased with time and that vibration may be more effective earlier. There was also a significant positive association between time since surgery and the magnitude of change in peak vGRF (R^2^ = 0.208, *p* < 0.01, *β* = 0.05). For every month post‐ACLR the effect of vibration on peak vGRF decreased by 0.05 xBW suggesting the effect of vibration reduced with time. Taken together, prolonged hamstring vibration appears to have the greatest impact on limb biomechanics early after surgery and may diminish with time. Baseline CAR had no association with the magnitude of change for any variable (Figure 4).

## 4 Discussion

We examined the effects of prolonged vibration of the hamstrings on limb stiffness during the loading phase of a single‐leg drop‐land task in individuals with ACLR and non‐injured controls. Increased stiffness is associated with smaller knee excursion and KEM and increased vGRF, which combine to increase loading rates. Therefore, we also determined the effects of prolonged hamstrings vibration on these associated variables. Consistent with our hypothesis, limb stiffness was lower in the ACLR group after vibration than before vibration; the ACLR group exhibited less peak vGRF and loading rates, and greater knee flexion excursion and KEM during the loading phase after versus before vibration. We also observed that the changes in biomechanics post‐vibration yielded outcomes that were statistically similar to controls. With the exception that while KEM increased after vibration, it remained lower in the ACLR group compared to controls. Finally, we observed a large effect of time since surgery with the pre‐ to post difference in both peak vGRF and stiffness. This suggests that prolonged vibration of the hamstrings has the potential to mitigate the stiff‐limb strategy commonly observe following ACLR, and may represent an effective adjunct therapy after ACLR, and that vibration may be most effective earlier in rehabilitation.

### 4.1 Stiffness

Diminished voluntary activation of the quadriceps causes muscle weakness [16], which results in a stiff‐limb loading strategy [6, 7, 16]. Increased hamstrings/quadriceps co‐activation may also contribute to diminished voluntary activation and quadriceps weakness [39] and it substantially stiffens the knee joint [55]. Some level of stiffness is needed for optimal performance [56]; however, too much over time can lead to knee OA [57, 58, 59]. Our findings show that prolonged hamstrings vibration can reduce limb stiffness in individuals with ACLR. This finding is consistent with previous studies that have shown that decreasing hamstrings/quadriceps co‐activation, and increasing quadriceps voluntary activation can lower stiffness after ACLR [44, 60].

The stiffer‐limb loading strategy observed in the present study may be a compensation mechanism to protect the ACL graft. Although greater co‐activation of the hamstrings and quadriceps is a protective response that limits anterior tibial translation, increases joint stability [61], and protects against excessive loading of the ACL, which is important in the early stage following injury, persistent leg stiffening as observed following ACLR, and in the participants of the present study who were more than 1 year post injury, can lead to negative changes in joint health, including larger compressive forces at the knee [52], cartilage thinning, and knee OA in patients with ACLR [62].

In the present study, there was a 14% reduction in lower limb stiffness following vibration. Lower limb stiffness is a function of peak vGRF and center of mass displacement. Peak vGRF was significantly lower after vibration than before vibration in the ACLR group. Knee flexion is a key factor for attenuating GRFs [63] and landing with greater knee flexion reduces landing forces [63]. After ACLR individuals appear to walk with smaller knee‐flexion angles than their uninjured counter‐parts [64]. Persistent reductions in knee‐flexion angle during jump landings have been linked to re‐rupture of the ACL [22], and smaller KEM and larger peak vGRF are associated cartilage compositional changes indicative of OA progression [12]. After vibration, there was 16% increase in knee flexion excursion; therefore, the reduction in peak vGRF may be due to greater knee flexion excursion. Together, increased knee excursion and lower vGRF resulted in less overall stiffness. It should be noted that there is a lack of evidence supporting that aberrant drop jump landing mechanics influence knee OA development and joint tissue health to a similar degree as aberrant repetitive loading. Therefore it is unlikely that a single drop landing, or a few drop landings, as observed in the current study, are influencing OA development, but rather thousands of loading cycles such as gait. However, the stiff limb strategy observed in the current study is representative of the stiff limb strategy observed during gait, different only in magnitude. We would expect similar improvement in stiffness to be observed during gait, however, future work should evaluate the effect of stiffness post‐ACLR during gait.

### 4.2 Loading Rate

An essential function of the quadriceps is attenuating GRFs. Quadriceps dysfunction inhibits this ability, resulting in impulsive loading [65]. Higher loading rates have been reported in the ACLR limb compared to contralateral and healthy control limbs [66, 67]. Similarly, higher loading rates have been reported in individuals with radiographic knee osteoarthritis compared to healthy controls. Greater loading rates can lead to cartilage degradation [68] and produce greater disruptions of cartilage structure and biosynthesis in animal and explant models than lower loading rates [69, 70]. Landing with increased knee flexion excursion lowers vGRF and loading rate [71], suggesting that the increase in knee flexion observed after prolonged vibration of the hamstrings may be responsible for the decrease in peak vGRF and loading rate.

### 4.3 Knee Excursion and KEM

Individuals who develop radiographic OA 5 years post‐ACLR have smaller sagittal plane moments during the first 2 years post‐ACLR compared to those that do not develop OA. In the current study, we observed a 16% increase in knee excursion and 23% increase in KEM after vibration. We have previously reported that hamstrings/quadriceps co‐activation is reduced after prolonged vibration of the hamstrings [39, 72]. Increased hamstrings/quadriceps co‐activation can substantially stiffen the joint making it more stable against unexpected changes in external load [55]. Over time, this has a detrimental effect on articular cartilage and leads to OA [73]. Greater hamstrings/quadriceps co‐activation is associated with lower knee flexion excursion and smaller KEM. Smaller KEM and peak knee flexion angles occur in individuals with radiographic OA 5 years post‐ACLR [13]. Greater changes in cartilage morphology are linked to smaller sagittal plane moments [12, 74]. Therefore, the observed increases in knee excursion and KEM are likely due to a lower hamstrings/quadriceps co‐activation ratio resulting reduced reciprocal inhibition of the quadriceps from the hamstrings. Results from jump‐training support the conclusion that the reductions in knee excursion and KEM were due to a reduction in co‐activation, where knee flexion excursion was 26% greater during the loading phase of a jump‐land task after ACLR after training that induced a reduction of hamstrings/quadriceps co‐activation [60].

Landing from a jump produces external knee‐flexion moments that act to flex the knee. Effective landing requires resisting these external knee flexion moments with an internal knee extension moment created by muscles that work eccentrically to absorb kinetic energy through the lower extremity [28]. Adequate quadriceps function is therefore of utmost importance for controlling knee flexion and for attenuating force during a jump landing. Persistent quadriceps weakness compromises knee joint stability and hinders the active restraints needed to protect against external loads, increasing the risk of injury and joint degeneration [6].

In the present study, both knee excursion and KEM were greater during the loading phase of the jump‐land task after vibration in individuals with ACLR. Greater quadriceps muscle voluntary activation is associated with greater KEM and knee flexion excursion in individuals with ACLR [7]. Increasing voluntary activation improves quadriceps performance [16], and lower extremity biomechanics [44]. Previously we found that prolonged hamstrings vibration increases voluntary activation of the quadriceps [39, 72]. Taken together, this suggests that the observed increases in KEM and knee excursion are due to increased voluntary activation of the quadriceps following prolonged vibration of the hamstrings. Which allows the quadriceps to better eccentrically control the knee and attenuate loading forces. It is also important to point out that changing knee flexion during the loading phase will likely change the cartilage contact and loading patterns, which may alter the cartilage biomechanics making it more susceptible to damage and degeneration [75]. Modifying lower limb biomechanics (i.e. increasing knee flexion after vibration) may improve tissue mechanics which may slow or postpone knee OA [76], but this remains unknown. Future studies should investigate if and how changing lower limb biomechanics changes cartilage loading.

Finally, during pre‐testing our cohort of ACLR participants displayed greater stiffness and associated variables (ie, smaller knee flexion excursion and KEM, and greater peak vGRF and instantaneous loading) compared to the NI controls. The results of the current study indicate that acute vibration has the potential to substantially improve limb loading mechanics and mitigate the stiff limb loading strategy. While the current study only evaluated the acute effects of prolonged hamstrings vibration. Repeated exposure to vibration embedded in ACLR rehabilitation has been shown to improve neuromuscular and postural control to a greater extent than rehabilitation alone [77, 78, 79]; and improve walking performance in individuals with knee OA [80].

Previously we demonstrated [72, 81], and others [82], that improvements in quadriceps function persist for up to an hour. This suggests that improvement in lower limb biomechanics, as a result of increased quadriceps activation, could last for at least an hour after vibration. Therefore, applying vibration at the beginning of a rehabilitation session may maximize the effect of rehabilitation protocols and provide a strategy to relearn healthy motor control patterns following ACLR, improving knee biomechanics and long‐term outcomes. Providing a viable adjunct therapy for improving ACLR rehabilitation. However, future research is necessary to evaluate the effects of repeated exposure, and embedded in rehabilitation, on gait biomechanics, joint health, and OA risk.

### 4.4 Limitations

There are limitations to our study that should be considered when interpreting the findings. First, we assessed only the acute effects of vibration. Thus, we cannot draw conclusions regarding the benefits of repeated use on reinjury, joint health or OA risk. Second, the focus of the current study was limb stiffness which is characterized by sagittal plane biomechanics; however, frontal plane biomechanics also plays an important role in reinjury and OA risk. Future studies should evaluate the effects of prolonged hamstrings vibration on frontal plane biomechanics. Third, we did not control for graft type; 11 participants received patella tendon autographs and 3 had allografts. In our previous work, we did not observe differences in magnitude of change in quadriceps voluntary activation or hamstring/quadriceps coactivation due to graft type, suggesting that prolonged hamstrings vibration is not influenced by graft type [39]. Fourth, lower limb biomechanics may be altered bilaterally [83]: however during the first 12 months post‐ACLR, both the ACLR and contralateral limbs demonstrate biomechanical differences compared with control limbs [83]. Differences between the contralateral and control limbs increase from 6 to 12 months post‐ACLR [83], suggesting the contralateral limb becomes more like the ACLR limb and may not serve as a proper control. Furthermore, prolonged muscle vibration has been shown to have a neuromuscular effect on the contralateral limb [84]. This would suggest that prolonged muscle vibration may improve the biomechanics in both limbs, making it difficult to detect changes following vibration. Therefore, for the current study we chose to compare the ACLR limb to a NI control limb before and after vibration to evaluate the effects of prolonged hamstring vibration on the ACLR limb and we only reported changes in the ACLR limb following our intervention. Future research should evaluate bilateral effects of vibration. Finally, we utilized a drop‐land task. While it is likely an athletic population that is getting exposed to high magnitudes and rate of loading (eg, jumping, running, etc) contribute to OA. Evidence suggests that OA development is likely due to repeated cyclical loading rather than discrete, high impact tasks [85, 86]. Therefore, future studies should evaluate the effects of prolonged hamstrings vibration on gait biomechanics.

## 5 Conclusion

The results of this study demonstrate that prolonged hamstrings vibration acutely reduces limb stiffness and instantaneous loading rate, as well as increases knee flexion excursion and KEM in individuals with ACLR. Therefore, prolonged vibration of the hamstrings has the potential to mitigate altered lower limb biomechanics linked with reinjury and knee OA development in patients post ACLR and may provide a strategy that could be implemented as an adjunct to standard rehabilitation to facilitate the restoration of normal symmetrical movements. Future research should include measures of repeated vibration exposure to evaluate the longitudinal effects of vibration combined with rehabilitation protocols.

## Author Contributions

Timothy Lowe, HH, Xuanliang Neil Dong and Lisa Griffin conceptualized the study design. Timothy Lowe collected and assembled the data, completed analysis and interpretation of data, and drafted the manuscript. All authors have critically reviewed, revised, and approved the manuscript. All authors have approved the study and manuscript, warrant that it is factual, have agreed to its submission, and have the right to publish.

## Conflicts of Interest

The authors declare no conflicts of interest.
